# Improving Image Quality in Computed Tomography-Guided Biopsy Using Deep Learning Reconstruction

**DOI:** 10.7759/cureus.87213

**Published:** 2025-07-03

**Authors:** Kazutoshi Tsunou, Hiroaki Ishii

**Affiliations:** 1 Department of Central Radiology, Japanese Red Cross Okayama Hospital, Okayama City, JPN; 2 Department of Radiology, Japanese Red Cross Okayama Hospital, Okayama City, JPN

**Keywords:** dose length product, filtered back projection, hybrid iterative reconstruction, image noise, interventional radiology, radiation exposure, volume ct dose index

## Abstract

Computed tomography (CT)-guided biopsy offers procedural safety and is widely used for the diagnosis of lesions. Recently, CT systems with deep learning reconstruction (DLR) have become available, offering enhanced noise reduction and the potential to reduce radiation exposure. However, DLR does not support real-time imaging in CT fluoroscopy (CTF), limiting its use in interventional procedures.

In this technical report, we investigate the image quality and reconstruction time of DLR in CT-guided biopsy using conventional (non-CTF) methods. Using a routine inspection phantom provided with the CT system, imaging was performed at setting standard deviation (SD) values of 20, 30, 40, and 50 Hounsfield units (HU); image noise and reconstruction time for three reconstruction methods (filtered back projection (FBP), hybrid iterative reconstruction (HIR), and DLR) were measured. SD was used as a quantitative measure of image noise.

The results showed that DLR had the lowest image noise, followed by HIR and FBP, and the higher the setting SD, the more pronounced the difference. This indicates that DLR can improve image quality while maintaining diagnostic image quality and reducing radiation exposure. However, DLR had the longest reconstruction time, exceeding 10 seconds to output six images.

Although DLR has obvious advantages in image quality and dose reduction, the associated delay in image reconstruction currently limits its use to procedures where real-time imaging is not essential. When real-time imaging is not required, DLR should be used in conjunction with conventional methods to reduce patient radiation exposure.

## Introduction

Computed tomography (CT)-guided biopsy is a minimally invasive diagnostic procedure used to manage lesions [1-5]. CT provides a three-dimensional view of the relationship between the lesion and surrounding tissues, making the procedure relatively safe [1-5].

CT-guided biopsy techniques are generally classified into two types: the conventional and CT fluoroscopy (CTF) methods [3-7]. In the conventional method, the needle is advanced toward the lesion by repeatedly acquiring conventional CT images. In contrast, the CTF method allows the needle to be guided using real-time CT images. The main drawback of the CTF method is the higher radiation exposure to the operator compared to the conventional method [6,7]. However, real-time CT imaging is particularly useful in lung biopsies, where respiratory motion can cause lesion displacement [6,7]. Kim et al. compared the number of puncture attempts and the procedure time between the two methods in CT-guided lung biopsy [7]. Their results showed that both the number of punctures and the procedure time were significantly lower with the CTF method. Additionally, complications occurred less frequently with CTF.

At our institution, the CT-guided biopsy procedure follows these four steps: a pre-procedural helical CT scan is performed to visualize the lesion and surrounding structures and to determine the optimal access route; after administering local anesthesia, the needle is inserted along the planned trajectory; CT or CTF images are obtained at several time points, and tissue samples are collected from the lesion under image guidance; after needle removal, a final CT scan is acquired to confirm the absence of complications.

Since 2020, CT systems equipped with deep learning reconstruction (DLR) have been introduced into routine clinical practice [8]. DLR is an image reconstruction technique that enhances image quality by applying a deep convolutional neural network [9]. DLR models are trained using high-quality CT images reconstructed with model-based iterative reconstruction (MBIR), allowing optimization of image parameters. The primary advantage of DLR is enhanced noise reduction. This improvement has two clinically relevant implications: first, it helps radiologists identify lesions more easily in routine practice; second, it allows for lower radiation exposure to patients [8-10].

Although DLR has the potential to reduce patient radiation exposure in CT-guided biopsies, it is currently not compatible with CTF. Therefore, when real-time imaging is unnecessary (e.g., musculoskeletal biopsy), we primarily use the conventional method, which supports DLR. This combination may improve image quality while minimizing radiation exposure. However, image reconstruction with DLR can be time-consuming.

In this technical report, we assess the image quality and reconstruction time of CT-guided biopsy using DLR and discuss its utility and limitations.

## Technical report

Phantom study

CT scanning was performed using a 320-slice Aquilion ONE GENESIS Edition (Canon Medical Systems, Tochigi, Japan). The scan parameters were as follows: tube voltage of 135 kVp, rotation time of 0.5 sec/rotation, detector collimation of 40 × 0.5 mm, scan field of view (SFOV) of 320 mm, and tube current set using automatic exposure control (AEC) with a target standard deviation (SD) [11]. The AEC settings were configured using the SD for FC03, which is a filtered back projection (FBP) reconstruction mode. A routine inspection phantom provided with the CT system was used, and imaging was performed at setting SD values of 20, 30, 40, and 50 Hounsfield units (HU) (set at a slice thickness of 5 mm). The phantom included inserts of various materials (air, delrin, acrylic, nylon, and polypropylene) embedded in a water-filled acrylic container for CT attenuation accuracy evaluation.

The reconstruction settings were as follows: FC11 (FBP); FC11 with Adaptive Iterative Dose Reduction 3D (AIDR3D) Standard as hybrid iterative reconstruction (HIR); and advanced intelligent Clear-IQ Engine (AiCE) Body Sharp Standard as DLR.

**Figure 1:**
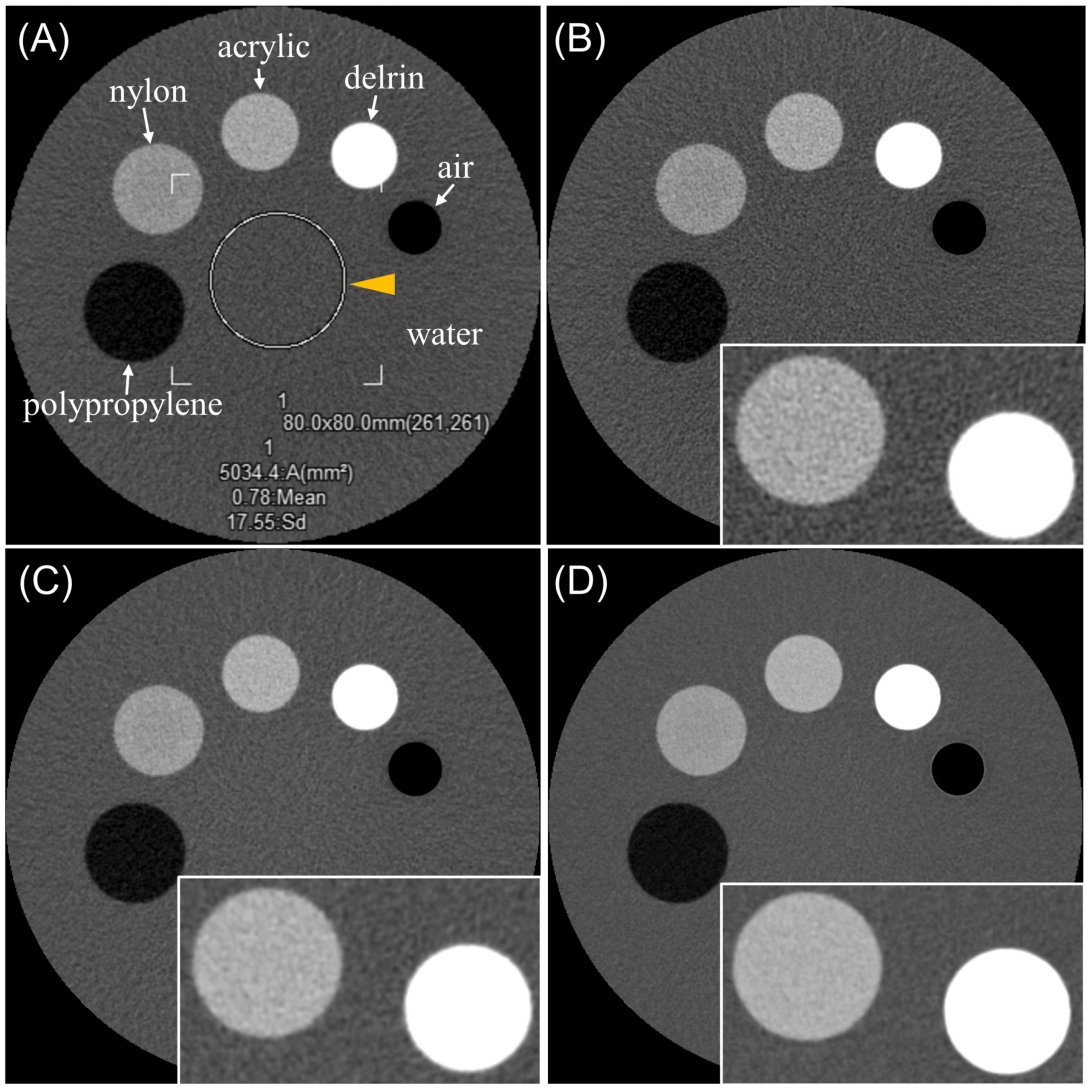
Overview of the phantom and SD measurements. (A) The phantom includes inserts made of different materials (air, delrin, acrylic, nylon, and polypropylene) placed in a water-filled acrylic container to evaluate CT attenuation accuracy. An 80 mm diameter ROI was placed at the center of the phantom, and the SD was measured. The yellow arrowhead indicates ROI. (B) Image reconstructed using FC11, a FBP method. (C) Image reconstructed using FC11 with AIDR3D Standard, representing HIR. (D) Image reconstructed using AiCE Body Sharp Standard, representing DLR. For (B), (C), and (D), the setting SD was 20 HU. Enlarged views of the delrin and acrylic regions are shown in the lower right corner of each image. SD: standard deviation; CT: computed tomography; ROI: region of interest; FBP: filtered back projection; AIDR3D: Adaptive Iterative Dose Reduction 3D; HIR: hybrid iterative reconstruction; AiCE: Advanced intelligent Clear-IQ Engine; DLR: deep learning reconstruction; HU: Hounsfield units

**Figure 2:**
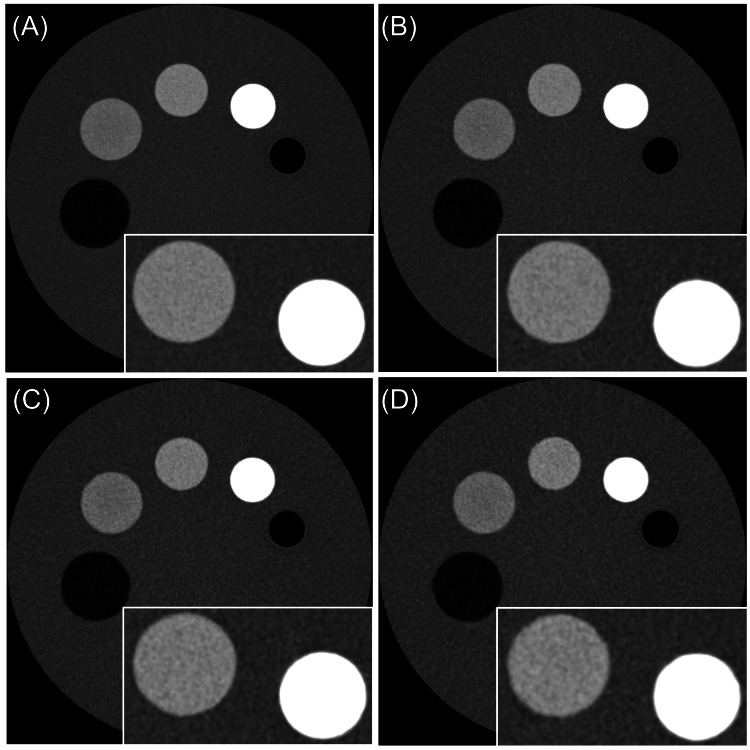
DLR images at different SD settings. (A) Setting SD: 20 HU; (B) Setting SD: 30 HU; (C) Setting SD: 40 HU; (D) Setting SD: 50 HU. DLR: deep learning reconstruction; SD: standard deviation; HU: Hounsfield units

Six 3.0 mm images were acquired for each setting. The phantom was positioned at the center of the gantry, and each scan condition was repeated five times. An 80-mm diameter region of interest (ROI) was set for SD measurements. Reconstruction time, measured from the end of image acquisition to completion of image reconstruction, was also evaluated at the setting SD 50 HU. Statistical analysis was conducted using EZR (Saitama Jichi Medical University, Saitama, Japan) [12]. Normality of continuous data was tested with the Shapiro-Wilk test. SD and reconstruction time differences were analyzed using paired t-tests with Bonferroni correction for multiple comparisons. A p-value < 0.05 was considered statistically significant. Figure 1 shows an example of the phantom and the reconstructed images at setting SD 20 HU. Figure 2 shows DLR images at setting SD 20, 30, 40, and 50 HU.

Phantom image quality and reconstruction time

Table 1 summarizes the SD and reconstruction time for each reconstruction image. Image noise was lowest with DLR, followed by HIR, and highest with FBP. The difference became more pronounced as the setting SD increased. Reconstruction time was fastest with FBP, followed by HIR, and slowest with DLR. Statistically significant differences in both SD and reconstruction time were observed among all comparisons (FBP vs. HIR, HIR vs. DLR, and FBP vs. DLR; p < 0.001).

**Table 1:** SD and reconstruction time for each reconstruction method The measured value indicates the SD (HU) of the image or the time required for reconstruction (sec). *Paired t-test with Bonferroni correction. SD: standard deviation; FBP: filtered back projection; HIR: hybrid iterative reconstruction; DLR: deep learning reconstruction; HU: Hounsfield units

Parameter		Measured value			p-value*
		FBP	HIR	DLR	
Setting SD	20 (HU)	17.74 ± 0.11	11.66 ± 0.11	9.08 ± 0.04	< 0.001
	30 (HU)	27.26 ± 0.18	14.52 ± 0.25	9.98 ± 0.13	< 0.001
	40 (HU)	33.24 ± 0.30	15.54 ± 0.18	10.22 ± 0.11	< 0.001
	50 (HU)	44.22 ± 0.85	16.78 ± 0.29	10.30 ± 0.16	< 0.001
Reconstruction time (sec)		1.69 ± 0.04	2.04 ± 0.07	10.34 ± 0.09	< 0.001

Clinical case

**Figure 3:**
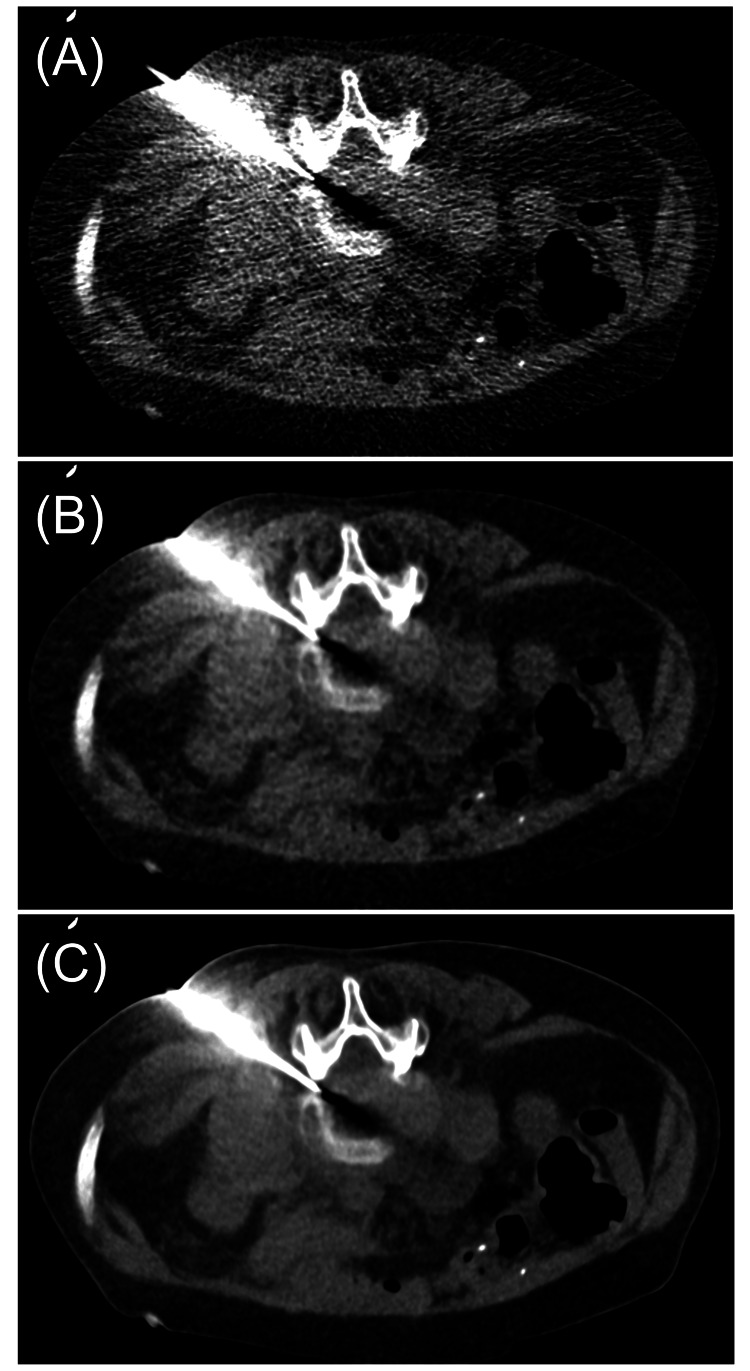
Clinical images from a CT-guided bone biopsy. Images obtained during the biopsy were reconstructed using the following methods: (A) Filtered back projection (FBP); (B) Hybrid iterative reconstruction (HIR); (C) Deep learning reconstruction (DLR).

A biopsy was performed on a woman in her 60s at the L4/L5 intervertebral disc. *Streptococcus agalactiae* was identified in the sample. No adverse events occurred following the procedure. The AEC setting during the biopsy was SD 50 HU. The dose length product (DLP) was 78.45 mGy·cm, and the volume CT dose index (CTDIvol) was 4.59 mGy. Reconstructed images under each condition from images taken during the biopsy are shown in Figure 3.

## Discussion

This technical report validated the image quality and reconstruction time of CT-guided biopsy using DLR.

The phantom study demonstrated that DLR provided the lowest SD and best image quality. The clinical images confirmed these findings. FBP showed the highest level of noise, which is problematic for image quality in CT-guided biopsy. HIR improved visibility of anatomical structures such as bones and needles, and DLR provided even greater noise reduction. Generally, in CT, reducing the radiation dose increases image noise. In CT-guided biopsy, noisy low-dose images may cause uncertainty in the procedure and require additional exposures, potentially increasing the patient's radiation exposure [4]. To reduce noise, it is necessary to increase the radiation dose per scan, which also increases the patient's radiation exposure. DLR offers superior noise reduction compared to both FBP and HIR, particularly in low-dose conditions [9,10]. Higaki et al. reported that DLR has higher lesion detection performance than FBP and HIR in low-dose CT, especially for structures with contrast levels of 50 to 200 HU, and that it is beneficial for soft tissue imaging such as abdominal CT [9]. Greffier et al. demonstrated that DLR could potentially reduce radiation doses by 9-56% while maintaining lesion detectability equivalent to HIR and DLR [13]. Moreover, under the same radiation dose, DLR improved the detection of low-contrast, small-diameter simulated lesions due to better noise texture and spatial resolution. These characteristics suggest that DLR is particularly advantageous for low-dose CT-guided biopsy. However, DLR had the slowest reconstruction time, taking more than 10 seconds to reconstruct six images.

Previous studies have shown the superiority of CT-guided interventional radiology procedures using DLR. Matsumoto et al. compared CT-guided interventional radiology procedures performed on systems with and without DLR [8]. In CT-guided interventional radiology procedures targeting biopsy, the DLP prior to intervention, after intervention, and in additional CT scans was significantly reduced in the DLR group compared to the non-DLR group. As a result, they reported that although there were no significant differences in success rates or complication rates, total DLP was reduced in the DLR group. However, in their study, DLR was not compatible with CTF, and no significant difference in radiation dose was observed between groups during CTF scans, regardless of the target organ. In this technical report, we aimed to reduce radiation exposure and improve image quality by using DLR in the conventional (non-CTF) method. In this technical report, since CTF scans were not used, DLR could be applied to all images. However, due to the delay in image reconstruction, DLR should be used only when real-time imaging is not required. For procedures requiring real-time imaging, CTF with HIR is recommended.

There are several limitations to this study. First, the evaluation was performed on a single CT system. Results may differ when using systems from other vendors or newer versions of the same system. Second, image quality was assessed only using SD; no evaluation of spatial resolution or subjective assessments by radiologists was conducted. Third, most of the validation was performed using a phantom. Future studies using clinical data to assess biopsy success rates and complication rates are warranted.

## Conclusions

This technical report examined the image quality and reconstruction time of CT-guided biopsy using DLR. DLR improved image quality while reducing patient radiation exposure. However, due to the time delay in image acquisition, DLR should be used with the conventional method when real-time imaging is not necessary, allowing for reduced patient radiation exposure.

## References

[1] Toki S, Sone M, Yoshida A (2022). Image-guided core needle biopsy for musculoskeletal lesions. J Orthop Sci.

[2] Devita R, Chagarlamudi K, Durieux J (2021). Omission of planning CT reduces patient radiation exposure during CT-guided bone marrow biopsy and aspiration. Tomography.

[3] Cahalane AM, Habibollahi S, Staffa SJ, Yang K, Fintelmann FJ, Chang CY (2023). Helical CT versus intermittent CT fluoroscopic guidance for musculoskeletal needle biopsies: impact on radiation exposure, procedure time, diagnostic yield, and adverse events. Skeletal Radiol.

[4] Sarti M, Brehmer WP, Gay SB (2012). Low-dose techniques in CT-guided interventions. Radiographics.

[5] Takaki H, Kobayashi K, Kako Y (2024). Computed tomography-guided puncture: preprocedural preparation, technical tips, and radioprotection. Interv Radiol (Higashimatsuyama).

[6] Froelich JJ, Ishaque N, Regn J, Saar B, Walthers EM, Klose KJ (2002). Guidance of percutaneous pulmonary biopsies with real-time CT fluoroscopy. Eur J Radiol.

[7] Kim GR, Hur J, Lee SM (2011). CT fluoroscopy-guided lung biopsy versus conventional CT-guided lung biopsy: a prospective controlled study to assess radiation doses and diagnostic performance. Eur Radiol.

[8] Matsumoto T, Endo K, Yamamoto S (2022). Dose length product and outcome of CT fluoroscopy-guided interventions using a new 320-detector row CT scanner with deep-learning reconstruction and new bow-tie filter. Br J Radiol.

[9] Higaki T, Nakamura Y, Zhou J, Yu Z, Nemoto T, Tatsugami F, Awai K (2020). Deep learning reconstruction at CT: phantom study of the image characteristics. Acad Radiol.

[10] Arndt C, Güttler F, Heinrich A, Bürckenmeyer F, Diamantis I, Teichgräber U (2021). Deep learning CT image reconstruction in clinical practice. Rofo.

[11] Funama Y, Awai K, Hatemura M, Shimamura M, Yanaga Y, Oda S, Yamashita Y (2008). Automatic tube current modulation technique for multidetector CT: is it effective with a 64-detector CT?. Radiol Phys Technol.

[12] Kanda Y (2013). Investigation of the freely available easy-to-use software 'EZR' for medical statistics. Bone Marrow Transplant.

[13] Greffier J, Hamard A, Pereira F, Barrau C, Pasquier H, Beregi JP, Frandon J (2020). Image quality and dose reduction opportunity of deep learning image reconstruction algorithm for CT: a phantom study. Eur Radiol.

